# Effect of chalcones in the modulation of *Trichophyton rubrum *cell wall synthesis genes

**DOI:** 10.1186/1753-6561-8-S4-P82

**Published:** 2014-10-01

**Authors:** Tamires Ap. Bittencourt, Juliana Simões Martins, Mariana Abreu, Tatiana Komoto, Luiz Felipe Bortolotto, Yasmim Crivelenti, Thais Mesquita, Vitor Pinhanelli, Bruna Cantelli, Mozart Marins, Ana Lucia Fachin

**Affiliations:** 1 Universidade de Ribeirão Preto, Ribeirão Preto, Brazil

## Background

*Trichophyton rubrum *is a dermatophyte that causes mostly superficial mycoses in skin, hair and nails, but an invasive course has been described in immunocompromised patients [1]. The resistance to usual antifungal drugs has been observed in *T.rubrum *what drives an increasing demand for new antifungal drugs. Chalcones are flavonoids found in plants that exhibit pronounced antifungal activity, most likely acting on the cell wall [2,3]. The aim of this study was to evaluate the modulation of expression of genes involved in cell wall synthesis of *T.rubrum *in the presence of chalcones.

## Methods

The *T. rubrum *strain H6 (ATCC MYA3108) was submitted to standard techniques for fungal manipulation and growth for 15 days as described previously by Fachin *et al*.[4]. The solution containing 5.10^6 ^conidia/ml of H6 *T.rubrum *strain was inoculated into 50 ml of liquid Sabouraud medium in the presence of 1.95 mg/ml of trans-chalcona metoxichalcona and controls aculeacin (0.24 mg/ml) and amphotericin (3.9 mg/ml) in a rotatory shaker at 28°C. After 8 hours of antifungal exposition, the RNA was extracted, converted into cDNA and was used in the experiments of real-time PCR. Expression levels were calculated by the comparative Ct method using 18S rRNA as normalizer gene and untreated mycelia as reference, according Bitencourt et al [5].

## Results and conclusion

The genes evaluated were DW699324 encoding the catalytic subunit of beta 1,3-glucan synthase in *Aspergillus fumigatus *[6]. The gene DW703091 (β-1,3-glucanosiltransferase) that is involved in the remodeling of 1,3-glucan in yeast [7]. The DW687782 is the gene encoding structural proteins of the membrane and the cell wall and is essential for the growth of *Candida albicans *[8]. The three genes of *T.rubrum *were induced in the presence of trans-chalcone, metoxichalcona and control aculeacin (acts on the wall) and were repressed in the presence of amphotericin (acts on the membrane). Thus, the antifungal effect of chalcones against *T.rubrum *may be related to modulation of these genes involved in cell wall synthesis.
